# Medical and Philosophical News

**Published:** 1781-03

**Authors:** 


### SECTION III.

### Medical and Philosophical News.

THE academy of ſciences at Dijon have propoſed the following ſubject for a prize of three hundred livres value: "To determine "with greater preciſion than hath been hitherto "done, the characteriſtic ſymptoms of intermit-"tent fevers, and to point out in the cleareſt "manner the circumſtances in which febrifuge "remedies may be employed with advantage, "and without danger to the ſick." The diſſertations on this ſubject are to be written in French or Latin, and ſent to M. Maret, ſecretary to the academy at Dijon, on or before the 1ſt of April 1782.

